# Supplementary material for: Socioeconomic inequalities in utilizing maternal health care in five South Asian countries: A decomposition analysis
Source: PLoS One. 2024 Feb 9;19(2):e0296762. doi: 10.1371/journal.pone.0296762 (PMC10857732; doi:10.1371/journal.pone.0296762)
Supplement: S1 Table — (DOCX) [file pone.0296762.s001.docx]

| **S1 Table.** Factors associated with ANC and institutional delivery: Bangladesh | | | |
| --- | --- | --- | --- |
| **Characteristics** | | **AOR ANC (95% CI)** | **AOR institutional delivery (95% CI)** |
| **Type of Place of Residence** | |  |  |
|  | Urban | 1.39 (1.20-1.60)*** | 1.31 (1.13-1.51)*** |
|  | Rural (RC) |  |  |
| **Maternal Age** | |  |  |
|  | 15-24 | 1.15 (0.87-1.53) | 1.13 (0.85-1.51) |
|  | 25-34 | 1.11 (0.84-1.46) | 0.97 (0.73-1.29) |
|  | 35-49 (RC) |  |  |
| **Body Mass Index** | |  |  |
|  | <18.50 (Underweight) | 0.73 (0.61-0.87)*** | 0.92 (0.77-1.09) |
|  | 18.50-24.90 (Normal) (RC) |  |  |
|  | 25.00-29.99 (Overweight) | 1.19 (1.01-1.40)* | 1.55 (1.30-1.84)*** |
|  | <30 (Obesity) | 1.16 (0.85-1.59) | 2.07 (1.45-2.95)*** |
| **Women Highest Education Level** | | |  |
|  | No education (RC) |  |  |
|  | Primary | 1.79 (1.31-2.45)*** | 1.29 (0.96-1.72) |
|  | Secondary | 2.75 (2.01-3.76)*** | 2.20 (1.64-2.95)*** |
|  | Higher | 3.54 (2.47-5.07)*** | 3.94 (2.78-5.58)*** |
| **Respondent Currently Working** | | |  |
|  | Not working (RC) |  |  |
|  | Working | 1.37 (1.20-1.56)*** | 0.82 (0.72-0.93)* |
| **Husband’s Education Level** | | |  |
|  | No education (RC) |  |  |
|  | Primary | 1.11 (0.90-1.36) | 1.06 (0.86-1.29) |
|  | Secondary | 1.39 (1.12-1.73)* | 1.20 (0.97-1.49) |
|  | Higher | 2.15 (1.65-2.82)*** | 1.85 (1.41-2.44)*** |
| **Occupation of the Husband** | |  |  |
|  | Agricultural (RC) |  |  |
|  | Non-Agricultural | 1.12 (0.95-1.32) | 1.16 (0.98-1.36) |
| **Wealth Status** | |  |  |
|  | Poorest (RC) |  |  |
|  | Poorer | 1.22 (1.01-1.48)* | 1.39 (1.15-1.68)** |
|  | Middle | 1.49 (1.22-1.82)*** | 1.80 (1.48-2.20)*** |
|  | Richer | 1.56 (1.27-1.92)*** | 2.29 (1.86-2.81)*** |
|  | Richest | 2.38 (1.87-3.03)*** | 3.86 (3.02-4.95)*** |
| **p<0.05; **p<0.01; ***p<0.001* | | | |
