# Supplementary material for: Socioeconomic inequalities in utilizing maternal health care in five South Asian countries: A decomposition analysis
Source: PLoS One. 2024 Feb 9;19(2):e0296762. doi: 10.1371/journal.pone.0296762 (PMC10857732; doi:10.1371/journal.pone.0296762)
Supplement: S2 Table — (DOCX) [file pone.0296762.s002.docx]

| **S2 Table.** Factors associated with ANC and institutional delivery: India | | | |
| --- | --- | --- | --- |
| **Characteristics** | | **AOR ANC (95% CI)** | **AOR institutional delivery (95% CI)** |
| **Type of Place 0f Residence** | |  |  |
|  | Urban | 1.13 (1.06-1.22)** | 1.10 (0.99-1.24) |
|  | Rural (RC) |  |  |
| **Maternal Age** | |  |  |
|  | 15-24 | 1.02 (0.93-1.12) | 1.69 (1.51-1.89)*** |
|  | 25-34 | 1.07 (0.98-1.16) | 1.34 (1.21-1.48)*** |
|  | 35-49 (RC) |  |  |
| **Body Mass Index** | |  |  |
|  | <18.50 (Underweight) | 1.03 (0.97-1.10) | 1.13 (1.04-1.22)* |
|  | 18.50-24.90 (Normal) (RC) |  |  |
|  | 25.00-29.99 (Overweight) | 1.16 (1.07-1.25)*** | 1.14 (1.02-1.28)* |
|  | <30 (Obesity) | 1.29 (1.13-1.49)*** | 1.39 (1.10-1.77)* |
| **Women Highest Education Level** | | |  |
|  | No education (RC) |  |  |
|  | Primary | 1.23 (1.12-1.35)*** | 1.13 (1.03-1.25)* |
|  | Secondary | 1.46 (1.35-1.57)*** | 1.68 (1.54-1.83)*** |
|  | Higher | 1.59 (1.42-1.78)*** | 3.59 (2.90-4.45)*** |
| **Respondent Currently Working** | | |  |
|  | Not working (RC) |  |  |
|  | Working | 1.11 (1.04-1.18)* | 0.89 (0.82-0.96)* |
| **Husband’s Education Level** | | |  |
|  | No education (RC) |  |  |
|  | Primary | 1.17 (1.06-1.29)* | 1.28 (1.16-1.42)*** |
|  | Secondary | 1.25 (1.15-1.36)*** | 1.50 (1.37-1.64)*** |
|  | Higher | 1.16 (1.03-1.30)* | 1.91 (1.61-2.27)*** |
| **Occupation of the Husband** | |  |  |
|  | Agricultural (RC) |  |  |
|  | Non-Agricultural | 1.04 (0.98-1.12) | 0.98 (0.89-1.08) |
| **Wealth Status** | |  |  |
|  | Poorest (RC) |  |  |
|  | Poorer | 1.31 (1.22-1.41)*** | 1.78 (1.64-1.92)*** |
|  | Middle | 1.82 (1.68-1.97)*** | 2.70 (2.44-3.00)*** |
|  | Richer | 2.14 (1.95-2.34)*** | 4.42 (3.82-5.10)*** |
|  | Richest | 2.44 (2.18-2.72)*** | 7.32 (5.85-9.16)*** |
| **p<0.05; **p<0.01; ***p<0.001* | | | |
