# Supplementary material for: Socioeconomic inequalities in utilizing maternal health care in five South Asian countries: A decomposition analysis
Source: PLoS One. 2024 Feb 9;19(2):e0296762. doi: 10.1371/journal.pone.0296762 (PMC10857732; doi:10.1371/journal.pone.0296762)
Supplement: S3 Table — (DOCX) [file pone.0296762.s003.docx]

| **S3 Table.** Factors associated with ANC: institutional delivery: Nepal | | | |
| --- | --- | --- | --- |
| **Characteristics** | | **AOR ANC (95% CI)** | **AOR institutional delivery (95% CI)** |
| **Type of Place 0f Residence** | |  |  |
|  | Urban | 1.23 (0.99-1.53) | 1.88 (1.55-2.27)*** |
|  | Rural (RC) |  |  |
| **Maternal Age** | |  |  |
|  | 15-24 | 1.19 (0.80-1.75) | 1.29 (0.88-1.89) |
|  | 25-34 | 0.99 (0.69-1.43) | 0.89 (0.62-1.28) |
|  | 35-49 (RC) |  |  |
| **Body Mass Index** | |  |  |
|  | <18.50 (Underweight) | 0.80 (0.61-1.06) | 0.75 (0.59-0.96)* |
|  | 18.50-24.90 (Normal) (RC) |  |  |
|  | 25.00-29.99 (Overweight) | 1.16 (0.82-1.62) | 1.51 (1.11-2.07)* |
|  | <30 (Obesity) | 1.49 (0.66-3.37) | 2.80 (1.17-6.72)* |
| **Women Highest Education Level** | | |  |
|  | No education (RC) |  |  |
|  | Primary | 1.76 (1.32-2.36)*** | 1.45 (1.11-1.89)* |
|  | Secondary | 2.71 (2.02-3.62)*** | 2.61 (2.01-3.38)*** |
|  | Higher | 8.14(4.81-13.79)*** | 4.67 (3.14-6.95)*** |
| **Respondent Currently Working** | | |  |
|  | Not working (RC) |  |  |
|  | Working | 1.46 (1.17-1.83)** | 1.03 (0.85-1.26) |
| **Husband’s Education Level** | | |  |
|  | No education (RC) |  |  |
|  | Primary | 1.23 (0.88-1.72) | 1.41 (1.03-1.93)* |
|  | Secondary | 1.36 (0.98-1.88) | 1.40 (1.04-1.90)* |
|  | Higher | 1.20 (0.77-1.88) | 1.31 (0.88-1.95) |
| **Occupation of the Husband** | |  |  |
|  | Agricultural (RC) |  |  |
|  | Non-Agricultural | 1.26 (0.97-1.63) | 1.25 (0.99-1.57) |
| **Wealth Status** | |  |  |
|  | Poorest (RC) |  |  |
|  | Poorer | 1.20 (0.89-1.62) | 1.08 (0.83-1.41) |
|  | Middle | 1.41 (1.04-1.92)* | 2.18 (1.66-2.86)*** |
|  | Richer | 1.78 (1.27-2.50)** | 2.80 (2.09-3.77)*** |
|  | Richest | 2.12 (1.28-3.52)* | 5.22 (3.22-8.45)*** |
| **p<0.05; **p<0.01; ***p<0.001* | | | |
