# Supplementary material for: Socioeconomic inequalities in utilizing maternal health care in five South Asian countries: A decomposition analysis
Source: PLoS One. 2024 Feb 9;19(2):e0296762. doi: 10.1371/journal.pone.0296762 (PMC10857732; doi:10.1371/journal.pone.0296762)
Supplement: S4 Table — (DOCX) [file pone.0296762.s004.docx]

| **S4 Table.** Factors associated with ANC: institutional delivery: Pakistan | | | |
| --- | --- | --- | --- |
| **Characteristics** | | **AOR ANC (95% CI)** | **AOR institutional delivery (95% CI)** |
| **Type of Place 0f Residence** | |  |  |
|  | Urban | 1.47 (1.22-1.78)*** | 1.19 (1.02-1.40)* |
|  | Rural (RC) |  |  |
| **Maternal Age** | |  |  |
|  | 15-24 | 1.10 (0.85-1.42) | 1.43 (1.16-1.77)** |
|  | 25-34 | 1.37 (1.10-1.70)* | 1.17 (0.98-1.40) |
|  | 35-49 (RC) |  |  |
| **Body Mass Index** | |  |  |
|  | <18.50 (Underweight) | 0.99 (0.72-1.38) | 1.00 (0.78-1.28) |
|  | 18.50-24.90 (Normal) (RC) |  |  |
|  | 25.00-29.99 (Overweight) | 1.24 (1.01-1.52)* | 0.97 (0.82-1.15) |
|  | <30 (Obesity) | 1.05 (0.82-1.35) | 1.44 (1.16-1.79)** |
| **Women Highest Education Level** | | |  |
|  | No education (RC) |  |  |
|  | Primary | 1.48 (1.15-1.90)* | 1.26 (1.02-1.55)* |
|  | Secondary | 2.33 (1.84-2.96)*** | 2.16 (1.74-2.68)*** |
|  | Higher | 4.35 (3.07-6.16)*** | 3.41 (2.44-4.77)*** |
| **Respondent Currently Working** | | |  |
|  | Not working (RC) |  |  |
|  | Working | 1.02 (0.77-1.36) | 1.02 (0.81-1.27) |
| **Husband’s Education Level** | | |  |
|  | No education (RC) |  |  |
|  | Primary | 1.32 (1.01-1.74)* | 1.43 (1.16-1.76)** |
|  | Secondary | 1.44 (1.14-1.82)* | 1.30 (1.09-1.56)* |
|  | Higher | 1.43 (1.07-1.91)* | 1.23 (0.97-1.56) |
| **Occupation of the Husband** | |  |  |
|  | Agricultural (RC) |  |  |
|  | Non-Agricultural | 0.94 (0.72-1.22) | 0.94 (0.77-1.14) |
| **Wealth Status** | |  |  |
|  | Poorest (RC) |  |  |
|  | Poorer | 1.44 (1.10-1.89)* | 1.38 (1.14-1.67)** |
|  | Middle | 2.39 (1.78-3.21)*** | 2.61 (2.08-3.27)*** |
|  | Richer | 2.51 (1.81-3.47)*** | 3.29 (2.52-4.30)*** |
|  | Richest | 6.76 (4.55-10.03)*** | 7.14 (4.98-10.25)*** |
| **p<0.05; **p<0.01; ***p<0.001* | | | |
