# Supplementary material for: Socioeconomic inequalities in utilizing maternal health care in five South Asian countries: A decomposition analysis
Source: PLoS One. 2024 Feb 9;19(2):e0296762. doi: 10.1371/journal.pone.0296762 (PMC10857732; doi:10.1371/journal.pone.0296762)
Supplement: Attachment — Submitted filename: Authors Response to Reviewers.docx [file pone.0296762.s005.docx]

**Authors’ Response to Reviewers’ Comments**

PONE-D-23-26546

**Socioeconomic Inequalities in Utilizing Maternal Health Care in Five South Asian Countries: A Decomposition Analysis**

We thank the editor and reviewers for their comments and appreciate the care taken in reviewing our manuscript. We hope that the revisions adequately address the concerns of the editor and reviewers. Below we provide our response to the editor’s and reviewers’ comments. All changes to the manuscript have been tracked in the file labeled ‘Revised Manuscript with Track Changes’.

|  | Reviewer’s Comment | Author’s Response |
| --- | --- | --- |
| **Reviewer #1** | Thanks to the authors for addressing an important topic and I am glad to review the paper. The paper is sound interesting and important, although lots of work already done on the same topic using secondary DHS dataset. | Thanks for your appreciation. |
|  | Please justify your argument why you research is important. | We have addressed your concerned. |
|  | I have some specific thoughts that needs to be addressed for publication and reaching to wider community. | Thanks. |
|  | Rationality of the paper is very week, please focus on the research gaps that is missing in the introduction section. | We have re-written the rationality |
|  | What is robustness of the analysis, why authors choose this statistical analysis? | We used the widely known and used statistical analysis to measure the inequality and factors associated with maternal health care utilisation, thus, we think that there is no question of the concentration curve analysis and elasticity analysis. In inequality measurements, decomposition methods are widely used. |
|  | Some grammatical errors found please check the whole manuscript. | All outstanding grammatical errors have been corrected. |
|  | What is the policy implications and theoretical contribution of this study? | the policy implications section revised. Page-17 |
|  | Why did you choose these five countries only while there are other South Asian countries have? | We have checked all the DHS data from South -Asia, and met these five countries |
|  | How you choose the independent variables for final model that is missing? | the dependent variables were chosen based on chi-square test of association whether the variables are associated with dependent variable or not. |
|  | What is power of concentration index and elasticity analysis? | Elasticity analysis can be applied in the context of income or wealth distribution to understand how changes in various economic factors (such as taxes, subsidies, wages, etc.) impact the distribution of income or wealth across different segments of the population. For example, income elasticity of demand can help analyze how changes in income levels affect the demand for certain goods or services, which can have implications for income distribution.  Additionally, elasticity analysis can be used to assess the responsiveness of income or wealth distribution to policy interventions aimed at reducing inequality. For instance, understanding the elasticity of taxable income with respect to changes in tax rates can help policymakers design tax policies that can potentially reduce income inequality.  Both the Power of Concentration Index and Elasticity Analysis provide valuable insights into the distribution of economic resources and how changes in economic variables affect inequality within a society. These analyses are essential for informed policy decisions aimed at mitigating inequality and promoting a more equitable distribution of resources. |
|  | I would suggest you to edit the conclusion based on the findings. | Conclusion and policy implications section have been rewritten. |
|  | Provide a calculation of explained and residual CIX in your decomposition table. | Correction have been made. |
|  |  |  |
| **Reviewer #2** | The objectives of this study were to assess socioeconomic disparities in the utilization of health care services by mothers and to determine the factors influencing this utilization among women aged 15 to 49 in five South Asian countries. This study has important policy implications. | Thanks for your acknowledgement. |
|  | The overall quality of this manuscript is good. | Thanks for your appreciation. |
|  | However, the quality will improve through linguistic and grammatical improvements. | All outstanding grammatical errors have been corrected. |
|  | Manuscript's similarity index should be checked to ensure uniqueness. | We have checked the similarity index and it is under 5% threshold. |
|  | The result section should be concise, reduce background characteristics. | thanks for your comments, since we have used five countries data therefore, results are bit larger, in addition, we compared the results among the countries thus is somewhat larger, however, we have tried our best to concise the results and background section too. |
|  | Would you please enrich the strengths and limitations section? | this section has been re-edited. |
