# Supplementary material for: Socioeconomic inequalities in utilizing maternal health care in five South Asian countries: A decomposition analysis
Source: PLoS One. 2024 Feb 9;19(2):e0296762. doi: 10.1371/journal.pone.0296762 (PMC10857732; doi:10.1371/journal.pone.0296762)
Supplement: Attachment — Submitted filename: Authors Response to editor.docx [file pone.0296762.s006.docx]

**Authors’ Response to Reviewers’ Comments**

PONE-D-23-26546

**Socioeconomic Inequalities in Utilizing Maternal Health Care in Five South Asian Countries: A Decomposition Analysis**

We thank the editor and reviewers for their comments and appreciate the care taken in reviewing our manuscript. We hope that the revisions adequately address the concerns of the editor and reviewers. Below we provide our response to the editor’s and reviewers’ comments. All changes to the manuscript have been tracked in the file labeled ‘Revised Manuscript with Track Changes’.

Additional Editor Comments:

- The paper addressed all the comments and now it can be acceptable after doing a minor change. Provide the values for sub-total of each variable in the decomposition tables of both outcome variables. Then give the summarized explained, and residual CIX contribution just beneath the “absolute contribution” column in Table 3 and Table 4.

Answer: This article has been substantially improved and the explained, and residual CIX provided in the tables below.
